# Alterations in Oral–Nasal–Pharyngeal Microbiota and Salivary Proteins in Mouth-Breathing Children

**DOI:** 10.3389/fmicb.2020.575550

**Published:** 2020-10-09

**Authors:** Cancan Fan, Lihong Guo, Haijing Gu, Yongbiao Huo, Huancai Lin

**Affiliations:** ^1^Hospital of Stomatology, Guanghua School of Stomatology, Sun Yat-sen University, Guangzhou, China; ^2^Guangdong Provincial Key Laboratory of Stomatology, Guangzhou, China

**Keywords:** microbiota, oral–nasal–pharyngeal, salivary protein, mouth breathing, high-throughput sequencing, oxidative stress

## Abstract

Mouth breathing induces a series of diseases, while the influence on microbiota of oral cavity and salivary proteins remains unknown. In this study, for the first time, profiles of oral–nasal–pharyngeal microbiota among mouth-breathing children (MB group, *n* = 10) were compared with paired nose-breathing children (NB group, *n* = 10) using 16S ribosomal DNA (rDNA) (V3–V4 region) high-throughput sequencing. The differentially expressed salivary proteins were revealed using label-free quantification (LFQ) method, and their associations with bacterial abundance were measured by canonical correspondence analysis (CCA). The overall bacterial profiles differed between the two groups, and the differences were related to the duration of mouth breathing. The diversity of oral–pharyngeal microbiota was significantly higher, and the nasal–pharyngeal species tended to be consistent (unweighted UniFrac, *p* = 0.38) in the MB group. Opportunistic pathogens were higher in relative abundance as follows: *Acinetobacter* in the anterior supragingival plaque, *Neisseria* in unstimulated saliva, *Streptococcus pneumoniae* in the pharynx, and *Stenotrophomonas* in the nostrils. The expression level of oxidative-stress-related salivary proteins (lactoylglutathione lyase and peroxiredoxin-5) were upregulated, while immune-related proteins (integrin alpha-M and proteasome subunit alpha type-1) were downregulated in MB group. The differentially expressed proteins were associated with specific bacteria, indicating their potentials as candidate biomarkers for the diagnosis, putatively early intervention, and therapeutic target of mouth breathing. This study showed that mouth breathing influences the oral–nasal–pharyngeal microbiota and enriches certain pathogens, accompanied with the alterations in the salivary environment. Further research on the pathological mechanisms and dynamic changes in longitudinal studies are warranted.

## Introduction

Microbiota and proteins play a very important role in human health and have a profound effect on human diseases. As the entry portal to the digestive and respiratory tracts, the oral cavity microbiome is strongly related to systemic disease (Liu, 2018), including inflammatory bowel disease (IBD), adverse pregnancy outcomes (APOs), rheumatoid arthritis (RA), human immune deficiency virus (HIV) infection, cardiovascular diseases, and Alzheimer’s disease (AD) (Gao et al., 2018). Respiratory tract flora are also associated with many diseases, such as asthma (Izuhara et al., 2016), cystic fibrosis (CF) (Pittman et al., 2017), chronic obstructive pulmonary disease (COPD) (Dima et al., 2016), pneumonia (Gollwitzer and Marsland, 2014), and lung cancer (Hosgood et al., 2014). Saliva could reflect physiological or pathological states of an individual (Loo et al., 2010). Thus, salivary proteins also have been suggested to be a diagnostic tool for oral and systemic diseases, such as oral leukoplakia, caries, periodontal disease, Sjögren’s syndrome, cancer, cardiovascular disease, and stroke (Castagnola et al., 2017; Lorenzo-Pouso Alejandro et al., 2018). Little is known, however, whether exogenous factors, such as mouth breathing, will cause dysbiosis of the microbiota or alteration of saliva state. Indeed, mouth breathing has attracted more considerable attention, with prevalence between 48 and 56.8% (Felcar et al., 2010; Limeira et al., 2013).

The human airways are important in heating and humidifying air during inhalation. Mouth breathers inspire and expire through the mouth, as a consequence of reduced patency of the nasal airways. The constant airflow from mouth breathing could dry the teeth and mucosa, especially in the anterior portion of the mouth (Pacheco et al., 2015), leading to chronic gingival inflammation (Sharma et al., 2016), an increased level of *Streptococcus mutans* (CFU > 10^5^), and a higher plaque index (PlI), although no significant difference was found in mean buffering capacity of saliva and the salivary flowrate (Mummolo et al., 2018). A higher risk of dental erosion and caries also exist among mouth breathers because of a decrease in intraoral pH compared with normal breathing during sleep (Choi et al., 2016). Mouth breathing may also lead to malocclusion (Paolantonio et al., 2019) and systemic diseases, such as asthma, gastrointestinal dysfunction, and sleep disorder (Flutter, 2006). The above studies involved the adverse clinical effects; however, little is known regarding the distinction of microbiota and salivary proteins between children with and without mouth breathing. In the aggravation of mouth breathing, adenotonsillar hyperplasia involved with pathogens and cell-mediated humoral immune response is the main cause of the above symptoms (Zautner Andreas, 2012; Ramos Vinícius et al., 2019). Several research results suggested the following physiological process: a change in oropharyngeal humidification and temperature was found in mouth breathers regardless of closed or open mouths (Fischer et al., 2017), and microbial communities of the nose, mouth, and throat were affected by spatial heterogeneity and environmental features and, in turn, generate additional spatial heterogeneity (Proctor and Relman, 2017). There are clear differences in the microbiota between the nasal and oral cavities in healthy adults (Bassis et al., 2014), and different oral sites may provide distinct environment for bacterial adhesion, survival, and growth (Yu et al., 2017). Accumulating evidence (Livnat et al., 2010; Abe et al., 2018) support the hypothesis that mouth breathing cause an alteration of microbial communities together with some pathological mechanisms based on environment and interactions between host and microbiota. The mechanisms and related biomarkers would play an important role in the diagnosis and putative early intervention or therapeutic target of mouth breathing among children. To achieve this objective, 10 paired subjects were recruited for the mouth-breathing (MB) group and the matched nose-breathing (NB) group. Eight sites of oral–nasal–pharyngeal cavity were sampled for 16S ribosomal DNA (rDNA) gene sequence analysis, and the label-free quantification (LFQ) proteomics method was used to detect the unstimulated saliva.

## Materials and Methods

### Study Subjects

This study was conducted in accordance with the Declaration of Helsinki, and the protocol was approved by the Ethics Committee of Guanghua School of Stomatology (Ethics number: 2019-20; date of approval: 2019–3–1). With verbal agreement from the children, the guardians signed the informed consent for inclusion before the children participated in the study, then completed a detailed questionnaire about medical history, lifestyle, and the dietary habits of the children. Sample size was according to a previous literature (Yu et al., 2017) and following the table look-up scheme of paired study [the minimum number of pairs was 10 when the efficiency of test (1–β) was set 0.8 with bilateral test (1:1)]. Ten mouth-breathing children (MB group) and 10 nose-breathing children (NB group) were respectively matched by age (differ within 6 months), gender, and caries status (with or without active caries).

*Inclusion criteria*. The MB group was confirmed according to the inclusion criteria (Table 1) that were integrated from a previous study (Pacheco et al., 2015). Three steps including questionnaire, visual with clinical assessment, and breathing test were implemented in order. Children in the MB group had laryngological examination in the otorhinolaryngologic department before and were diagnosed as rhinitis/nasosinusitis and/or adenoidal hypertrophy.

**TABLE 1:** Inclusion criteria of mouth breathing children.

	Clinical examination and testing	Inclusion criteria
Questionnaire	a. Day conditions Lack of lip seal/breathing with mouth open/mastication with mouth open	In line with at least two items for a or b
	b. Night conditions Snoring/sleeping with mouth open/dry mouth in the next morning	
Visual assessment	Lip incompetency/high narrow palate/narrow dental arch/maxillary protrusion/mandibular retraction/crowded dentition	In line with at least one item
Breathing test	Water retention test, single nose test, mirror test, cotton batting test	In line with at least one item

*Exclusion criteria*. Children had taken antibiotics in the past 1 month; children with syndromes or chronic systemic diseases, craniofacial deformity, complete nostril obstruction and perforation of the nasal septum, or any known acute disease of the nasal or oral cavity, nasal sinuses, salivary glands, or pharynx were excluded (Ramos Vinícius et al., 2019).

### Sample Collection

**FIGURE 1:**
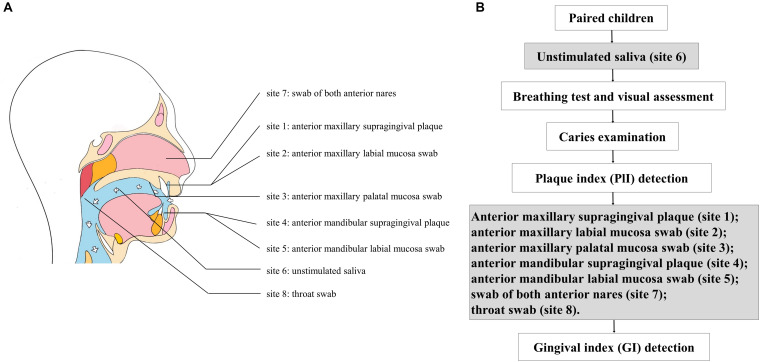
**(A)** Sampling of eight sites from each subject; **(B)** flow diagram of sampling and examination.

Samples were obtained from eight sites of each subject in the morning between 8:00 and 10:00 AM, as this is the most stable timepoint during the day for saliva composition (Dawes, 1972). Children were required to avoid diet or brushing for all night until sampling in the morning. The overall sampling and examination process are shown in Figure 1. Unstimulated saliva samples (site 6) were collected first. Children were instructed to sit and lean forward in a friendly dental clinic, rinse the mouth completely with pure water from dental chair supply at room temperature, and wait for 10 min, then expectorate into sterile tubes for 5 min. The other seven sites of samples were collected, including supragingival plaque (site 1) and mucosa swabs (labial and palatal, sites 2 and 3, respectively) of the anterior maxillary region, supragingival plaque (site 4) and labial mucosa swab (site 5) of the anterior mandibular region, swabs from both anterior nares (site 7), and throat swabs (site 8) (Figure 1A). The sampling procedures were following the Human Microbiome Project^1^; samples of saliva, soft tissue sites, and hard tissue sites were collected in sequence and labeled with appropriate preprinted label. Tubes were restored in a Ziploc bag and placed over ice and were transported to the laboratory in 1 h for further processing. After being centrifuged at 4,000 rpm (5,943 *g* at 4°C for 20 min), the saliva samples were isolated as sediments and supernatants, then saved at −80°C until use.

Dental examination was performed in dental chair under artificial light. The caries condition (decayed, missing, and filled tooth index, dmft/DMFT for primary/permanent teeth) and periodontal condition [plaque (PlI) and gingival (GI) index] were also recorded according to the methods described in the 5th edition of the WHO Oral Health Surveys—Basic Methods (World Health Organization, 2013). The PlI was determined before sampling, and the GI was detected after sampling to avoid possible pollution of bleeding (Figure 1B). Children with a decayed index >1 were considered to have caries; the PlI and GI of the anterior and posterior areas were calculated. The above clinical investigations and records were implemented by a trained examiner (CF). The intraexaminer kappa value of caries assessment was 0.86.

### Sample Processing and High-Throughput 16S rDNA Sequencing

The total genomic DNA was extracted from samples using a QIAamp DNA microkit (Qiagen, Germantown, MD, United States). The V3–V4 hypervariable regions of the bacterial 16S rDNA gene were amplified using universal 16S rDNA primers (341F: CCTACGGGNGGCWGCAG; 806R: GGACTACHVGGGTATCTAAT). The products of PCR amplification were collected by gel cutting and quantified using an ABI StepOnePlus Real-Time PCR System (Life Technologies, Foster City, CA, United States). The purified amplification products were pooled in equimolar and paired-end sequenced (2 × 250) on an Illumina platform (Hiseq2500 PE250), following the procedure used in the Human Microbiome Project (Human Microbiome Project Consortium, 2012). Poor-quality sequences were excluded using the default parameters of the Quantitative Insights into Microbial Ecology program (QIIME) script split_libraries.py (minimum average quality score = 25, minimum/maximum sequence length = 200/1,000 base pairs, no ambiguous base calls, and no mismatches allowed in the primer sequence). Filtered sequence reads were clustered into operational taxonomic units (OTUs) and subsequently assigned to taxa by the SILVA database of reference sequences with ≥97% identity. The representative sequences were classified into organisms by a naive Bayesian model using RDP classifier (version 2.2) based on the SILVA database (version 132) with an 80% confidence level by MOTHUR.

### Salivary Protein Detection With LFQ

Briefly, the supernatants of 20 saliva samples were added with L3 lysis buffer and 1% phenylmethylsulfonyl fluoride (PMSF) to extract the total proteins, and the concentration was determined using the Bradford assay. After reduction with 0.05 M tris (2-carboxyethyl) phosphine (TCEP) for 60 min and cysteine blocked with 55 mM methyl methanethiosulfonate (MMTS) for 45 min, the protein solution was added to a 10-kDa ultrafiltration tube (Pall Corporation, Port Washington, NY, United States) and digested with trypsin in 0.5 M triethylammonium bicarbonate (TEAB) (1:100 w/w) overnight at 37°C. Each peptide sample was vacuum dried using a Speedvac and subsequently dissolved with a buffer [0.1% formic acid (FA), 2% acetonitrile (ACN)] and centrifuged at 13,200 rpm for 20 min at 4°C. Peptides were identified with Thermo Scientific Q Exactive (Thermal Scientific, Chelmsford, MA, United States). The scan of first-grade mass spectrometry (MS) ranged from 350 to 1,800 m/z at a resolution of 70,000 and an automatic gain control (AGC) target of 3 × 10^6^. The scan of second-grade MS was initiated as 60 m/z at a resolution of 17,500 and an AGC target of 10^5^. For data from the Q-Exactive instrument, MS tolerance was set at 20 ppm and MS/MS tolerance at 0.5 Da. Three biological replicates were used for proteomic analyses. The mass spectrometry proteomics data have been deposited to the ProteomeXchange Consortium via the PRIDE partner repository with identifier PXD021106^2^.

The raw files from the mass spectrometer were transferred to Mascot Generic Files (MGF) and retrieved in the database downloaded from uniprot through Maxquant software (uniprot_rat_29389_20200312.fasta, included 29,389 sequences, downloaded in 2020–03–12). The liquid chromatography–tandem MS (LC-MS/MS) data were normalized and aligned according to the manufacturer’s specifications. The false discovery rate (FDR) was evaluated using the uniprot_rat library, and the proteins identified by sites, reverse database, and common contaminant database were filtered. Based on the fundamental analysis of protein and peptides, the differentially expressed proteins were screened (fold-change, 1.5; *p* < 0.05). Proteins from the LFQ experiment were processed with MetaCore to build an analysis of functional ontologies including canonical pathway maps. Functional annotation and pathway of the differentially proteins were performed to identify enriched cellular components, biological processes, and molecular functions between paired groups using gene ontology (GO) enrichment analysis.

### Statistical Analysis

The taxonomic composition and clinical characteristics of two groups were compared using non-parametric Wilcoxon rank-sum test. According to the diversity indices, α diversity (within-subject diversity) using ACE, Sobs, Chao1, Shannon, Good’s coverage, numbers of observed species (richness), and the Simpson’s Index (evenness) was assessed. β diversity (between-subject diversity) was analyzed via UniFrac (unweighted and weighted) and principle coordinate analyses (PCoAs) using Quantitative Insights into Microbial Ecology program (QIIME) (Caporaso et al., 2010) and displayed using the R software. Normality of the data distribution was evaluated with the Kolmogorov–Smirnov test. *p* < 0.05 were considered significant after adjustment for multiple tests.

Three steps of comparison were carried out for the microbial sequencing results: eight sites of samples were compared respectively between two groups, the six oral samples were merged as a whole and compared with nasal–pharyngeal samples, and the six oral samples were compared with each other, respectively. The differences within/between the MB and NB groups among oral–nasal–pharyngeal samples were evaluated using a Kruskal–Wallis test and a non-parametric Wilcoxon rank-sum test. A *p* < 0.05 was considered statistically significant. The area under curve (AUC) for each receiver operating characteristic (ROC) curve were calculated to assess the ability to differentiate two groups as candidate biomarkers.

A unimodal model [canonical correspondence analysis (CCA)] was constructed to evaluate the effect of differentially expressed protein, duration of mouth breathing, and clinical characteristics (age and gender of participants, with or without caries, and the PLI and GI) on microbiota composition.

## Results

One hundred sixty samples from 8 sites of 10 mouth-breathing and 10 paired nose-breathing children were analyzed for this study. We obtained a total of 13,268,156 high-quality bacterial 16S rDNA sequences. The sequences were clustered into 46,880 operational taxonomic units (OTUs) with 97% identity. Overall, 34 phyla, 89 classes, 178 orders, 292 families, and 601 genera were annotated from the 46,880 OTUs. The general information and clinical characteristics of paired groups are exhibited in Table 2. Among the participants, three paired children had caries, and seven paired children were caries-free. The PlI and GI of the anterior (A) and posterior (P) areas were calculated and compared in MB group that were PlI (A): 1.53 ± 0.49, PlI (P): 1.42 ± 0.51, GI (A): 1.36 ± 0.35, GI (P): 1.28 ± 0.27; and in NB group that were PlI (A): 1.32 ± 0.39, PlI (P): 1.40 ± 0.31, GI (A): 1.22 ± 0.19, GI (P): 1.28 ± 0.25; the MB group had slightly higher PlI and GI index than the NB group, especially in anterior area, but the differences were not significant (*p* = 0.31, 0.92, 0.29, 0.99, respectively).

**TABLE 2:** Clinical characteristics of 10 paired subjects^a^.

Subject ID	Gender M/F	Age	dt (Dt)/mt (Mt)/ft (Ft)	PlI A/P	GI A/P	Duration of mouth breathing	History of nasopharyngeal diseases
A1	M	10 years	0/0/7	2.00/1.75	1.83/1.33	3–5 years	Rhinitis/nasosinusitis
B1	M	10 years	0/0/2	1.33/1.42	1.17/1.25	–	No
A2	F	8 years	0/0/0	1.27/1.08	1.08/1.25	3–5 years	Rhinitis/nasosinusitis, adenoidal hypertrophy
B2	F	8 years	0/0/7	1.08/1.17	1.17/1.00	–	No
A3	F	9 years	3/0/4	1.58/1.58	1.58/1.00	1–2 years	Adenoidal hypertrophy
B3	F	9 years	1/0/5	1.17/1.83	1.25/1.58	–	No
A4	M	14 years	0/0/0	0.58/0.58	0.83/1.25	>5 years	Rhinitis/nasosinusitis
B4	M	14 years	0/0/3	1.42/1.25	1.67/1.33	–	No
A5	M	8 years	0/0/8	1.83/2.17	1.25/1.08	1–2 years	Rhinitis, antiadoncus, adenoidal hypertrophy
B5	M	8 years	0/0/4	0.83/1.17	1.17/1.00	–	No
A6	M	10 years	0/0/3	1.50/1.00	1.10/1.00	>5 years	Rhinitis/nasosinusitis
B6	M	10 years	0/2/6	2.17/2.00	1.08/1.08	–	No
A7	F	10.5 years	0/0/8	2.4/2.08	1.5/1.58	3–5 years	Rhinitis/nasosinusitis
B7	F	10.5 years	0/0/0	1.75/1.58	1.42/1.42	–	No
A8	F	7 years	2/0/0	1.64/1.58	1.83/1.67	3–5 years	Rhinitis/nasosinusitis, antiadoncus, adenoidal hypertrophy
B8	F	7 years	5/0/6	1.40/1.13	1.00/1.75	–	No
A9	M	8 years	0/0/0	1.17/1.25	1.58/1.67	1–2 years	Rhinitis/nasosinusitis, adenoidal hypertrophy
B9	M	8 years	0/0/4	1.08/1.18	1.08/1.18	–	No
A10	M	7 years	7/0/1	1.33/1.08	1.00/1.00	3–5 years	Rhinitis/nasosinusitis
B10	M	7 years	5/0/6	1.00/1.25	1.20/1.25	–	No

*^a^A represent the MB group, B represent the NB group; dt (Dt), decayed tooth; mt (Mt), missing tooth; ft (Ft), filled tooth; A/P, anterior/posterior area.*

### Taxonomic Composition of the Mouth- and Nose-Breathing Group Samples

**FIGURE 2:**
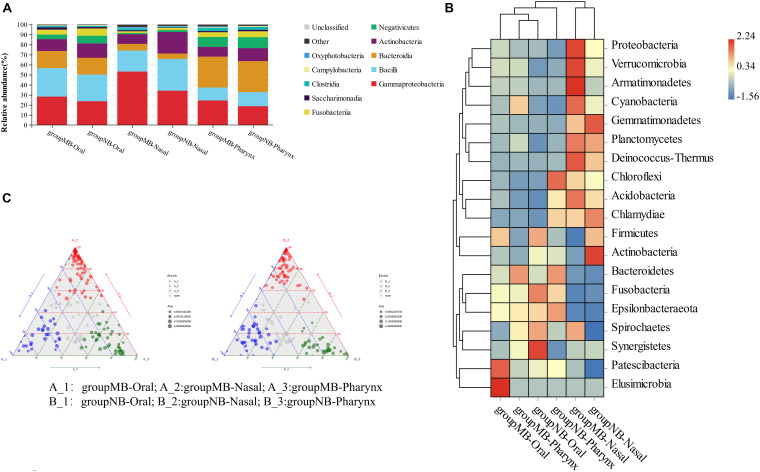
**(A)** Relative abundance (%) of bacterial community compositions at class levels; **(B)** heatmap analysis at phylum level; **(C)** ternary plots at genus level in oral–nasal–pharyngeal samples of the mouth-breathing (MB) and nose-breathing (NB) groups.

Among children in the two groups, the dominant bacteria and abundance of oral–nasal–pharyngeal samples were different. The most abundant genera of the oral cavity in the MB group were *Streptococcus*, *Neisseria*, *Haemophilus*, *Alloprevotella*, *Actinomyces*, and *Gemella*. The dominant genera of nasal samples (site 7) in the MB group were *Haemophilus*, *Moraxella*, *Streptococcus*, *Acinetobacter*, *Pseudomonas*, and *Neisseria*. The dominant genera of pharyngeal samples (site 8) in the MB group were *Neisseria*, *Prevotella_7*, *Streptococcus*, *Veillonella*, *Alloprevotella*, and *Actinomyces*. The most abundant genera of the six sites from oral samples in both groups were *Neisseria* (sites 1, 4, and 6) and *Streptococcus* (sites 2, 3, and 5). The relative abundance of the bacterial community, heat map of species abundance, and ternary plots at the genus level among oral–nasal–pharyngeal samples between the MB and NB groups are shown in Figure 2. Distinctions in taxonomic composition between the two groups were shown.

The significantly different species among oral–nasal–pharyngeal samples between groups were screened with an AUC value higher than 0.7. In oral samples, increased *Acinetobacter calcoaceticus* (0.787) and *Escherichia–Shigella* (0.712) and decreased *Olsenella* (0.758) had power to distinguish the MB group. In nasal samples, increased *A. calcoaceticus* (0.820), *Pseudomonas* (0.820), *Stenotrophomonas* (0.920), *Bacteroides* (0.815), *Faecalibacterium* (0.830), *Lysobacter* (0.820), and *Ralstonia* (0.850), and decreased *Capnocytophaga* sp. oral taxon 863 str F0517 (0.785) characterized the MB group. In pharyngeal samples, there was increased *A. calcoaceticus* (0.865), *Ruminococcus_2* (0.770), *Streptococcus pneumoniae* (0.770), and *Dialister pneumosintes* (0.780) and decreased *Corynebacterium* (0.790) and *Olsenella* (0.765) in the MB group. *A. calcoaceticus* was significantly increased in the oral–nasal–pharyngeal samples, and *Olsenella* was significantly decreased in the oral–pharyngeal samples of the MB group, and increased *Stenotrophomonas* in the nasal sample had a strong predictive power to distinguish the MB group.

### Differences in Microbiota Diversity Among Oral–Nasal–Pharyngeal Samples

#### Eight Sites Comparison

For the eight sites of microbial samples, significant differences existed between the two groups based on α and β diversity analysis. The Wilcoxon test showed that the site 8 sample (*p* = 0.03) had a significantly higher index of Chao1 in the MB group than the NB group, illustrating that the microbial community richness in the pharynx increased significantly. The unweighted UniFrac index of samples, including sites 1, 3, 4, 5, and 8 between the two groups, were significantly different (*p* < 0.001). The weighted UniFrac index of sites 1 (*p* = 0.01), 2 (*p* = 0.04), 4 (*p* < 0.001), and 6 (*p* = 0.01) samples were significantly different. The results indicated that the species in the anterior maxillary mucosa swabs (site 2) and unstimulated saliva (site 6) between the two groups were nearly the same but with a different richness. Two sites of samples [sites 1 and 4 (anterior supragingival plaque)] were significantly different in both microbial species and richness between the two groups.

#### Oral–Nasal–Pharyngeal Comparison

**FIGURE 3:**
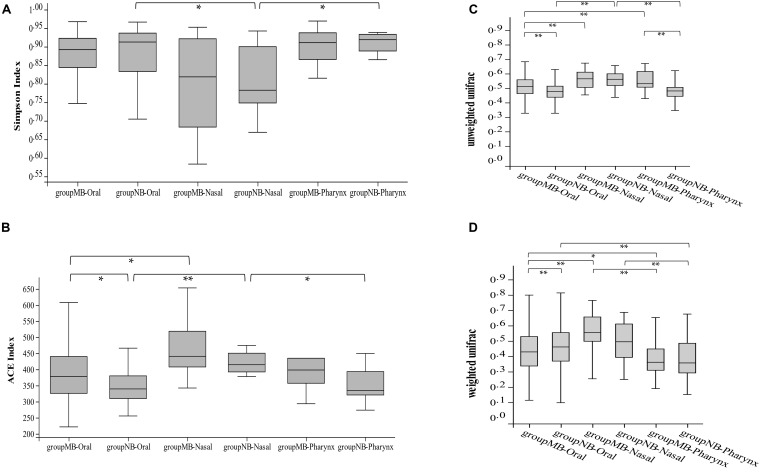
The comparison of **(A)** Simpson and **(B)** ACE indices of bacterial community of oral–nasal–pharyngeal samples among the mouth-breathing (MB) and nose-breathing (NB) groups; the comparison of **(C)** unweighted UniFrac and **(D)** weighted UniFrac analysis of bacterial community of oral–nasal–pharyngeal samples among the MB and NB groups; *p < 0.05, **p < 0.001.

We merged the six sites of oral samples as a whole and compared with nasal and pharyngeal samples by α diversity to study the microbial evenness and richness within samples. The analysis showed significant differences among the oral cavity, nasal cavity, and pharynx in the NB group (Simpson index, Kruskal–Wallis test, *p* = 0.01), while there is no statistical difference in the MB group (Simpson index, Kruskal–Wallis test, *p* = 0.17). The Wilcoxon test showed that oral samples had significantly higher ACE (*p* = 0.002) and Chao1 (*p* = 0.01) indices, and pharyngeal samples had a higher Chao1 index (*p* = 0.03) in the MB group than in the NB group. There were no differences between the nasal and pharyngeal samples in the MB group (Simpson, *p* = 0.11). Comparisons of the Simpson and ACE indices of the bacterial community in the oral–nasal–pharyngeal samples among the MB and NB groups are shown in Figures 3A,B.

The unweighted and weighted UniFrac calculations showed differences among the three cavity samples in both groups (Kruskal–Wallis test, *p* < 0.001). The unweighted UniFrac calculation showed species consistency among the pharyngeal and oral samples in NB group (*p* = 0.46) changed to consistency among nasal and pharyngeal samples in the MB group (*p* = 0.41). The oral (*p* < 0.001) and pharyngeal (*p* < 0.001) samples had significant differences between two groups (Figures 3C,D). Higher bacterial diversity of the oral–pharyngeal samples and the species consistency of the nasal–pharyngeal samples existed in the MB group.

#### Six Sites of Oral Samples

**FIGURE 4:**
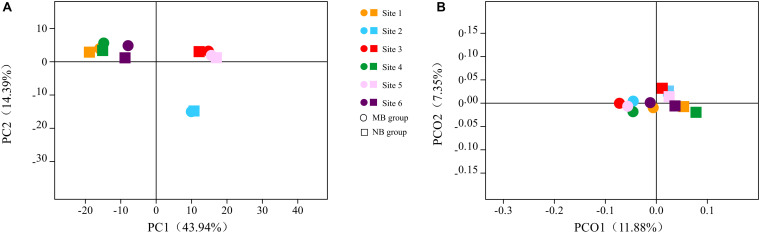
The **(A)** principal component analysis (PCA) and **(B)** principle coordinate analysis (PCoA) based on unweighted UniFrac analysis of six oral samples in two groups.

A comparison of the six oral sample sites also yielded meaningful results. The principal component analysis (PCA) of samples is shown in Figure 4A; different oral sites harbored distinct species. The α diversity analysis showed significant differences among the six sites of samples in the NB group (Good’s coverage, Kruskal–Wallis test, *p* = 0.03), while no significant difference was detected in the MB group (Good’s coverage, Kruskal–Wallis test, *p* = 0.06). The unweighted UniFrac analysis showed significant differences between site 2 and site 3/4/5 in the NB group (*p* < 0.001) and no differences between sites 2 and 3 (*p* = 0.59)/site 4 (*p* = 0.44)/site 5 (*p* = 0.39) in the MB group. Weighted UniFrac analysis showed differences between sites 4 and 3 (*p* = 0.004)/site 6 (*p* = 0.002), sites and 2 (*p* = 0.009) in the NB group and no differences between sites 4 and 3 (*p* = 0.39)/site 6 (*p* = 0.13), sites 1 and 2 (*p* = 0.14) in the MB group. The PCoA analysis of unweighted UniFrac is shown in Figure 4B; the overall species of the six sites of oral samples were nearly the same within groups but different between the two groups. With respect to the indicator species, the relative abundance of *Acinetobacter* was higher in sites 1 and 4 (anterior supragingival plaque), *Aggregatibacter* was lower in site 3 (anterior maxillary palatal mucosa), and *Neisseria* was higher in site 6 (unstimulated saliva) in the MB group compared with the NB group.

### The LFQ of Salivary Proteins

In analyzing the collected saliva samples with the LFQ approach, 740 proteins and 4,744 peptides were identified and used for further comparative quantification (FDR < 0.1% at the protein level). Ten proteins from our LFQ data were differentially expressed between the MB and NB groups with a fold change >1.5 (Table 3). Five of these proteins had upregulated expression, and the other five proteins were downregulated in the MB group. Although the abundance of most proteins was not significantly altered between the MB and NB groups, the levels of lactoylglutathione lyase (GLOl) and peroxiredoxin-5 (PRDX5) were significantly higher (2.04- and 1.62-fold, respectively; *p* = 0.03), while integrin alpha-M [ITGAM (0.66-fold); *p* = 0.01] and proteasome subunit alpha type-1 [PSMA1 (0.52-fold); *p* = 0.02) were significantly lower in the MB group compared with the NB group.

**TABLE 3:** The differentially expressed salivary proteins of two groups.

Accession	Protein name	Mass (kDa)	Fold change (B/NB)	*p* value
P27482	Calmodulin-like protein 3 OS = *Homo sapiens* OX = 9,606 GN = CALML3 PE = 1 SV = 2	16.89	1.52	0.07
Q96S96	Phosphatidylethanolamine-binding protein 4 OS = *Homo sapiens* OX = 9,606 GN = PEBP4 PE = 1 SV = 3	25.73	1.51	0.10
P30044	Peroxiredoxin-5, mitochondrial OS = *Homo sapiens* OX = 9,606 GN = PRDX5 PE = 1 SV = 4	22.08	1.62	0.03
P48594	Serpin B4 OS = *Homo sapiens* OX = 9606 GN = SERPINB4 PE = 1 SV = 2	44.85	0.44	0.06
Q9BRK5	45 kDa calcium-binding protein OS = *Homo sapiens* OX = 9,606 GN = SDF4 PE = 1 SV = 1	41.80	0.62	0.13
Q05315	Galectin-10 OS = *Homo sapiens* OX = 9,606 GN = CLC PE = 1 SV = 3	16.45	1.60	0.14
P11215	Integrin alpha-M OS = *Homo sapiens* OX = 9,606 GN = ITGAM PE = 1 SV = 2	127.18	0.66	0.01
P25786	Proteasome subunit alpha type-1 OS = *Homo sapiens* OX = 9,606 GN = PSMA1 PE = 1 SV = 1	29.55	0.52	0.02
Q04760	Lactoylglutathione lyase OS = *Homo sapiens* OX = 9,606 GN = GLO1 PE = 1 SV = 4	20.77	2.04	0.03
Q14974	Importin subunit beta-1 OS = *Homo sapiens* OX = 9,606 GN = KPNB1 PE = 1 SV = 2	97.17	0.44	0.11

**FIGURE 5:**
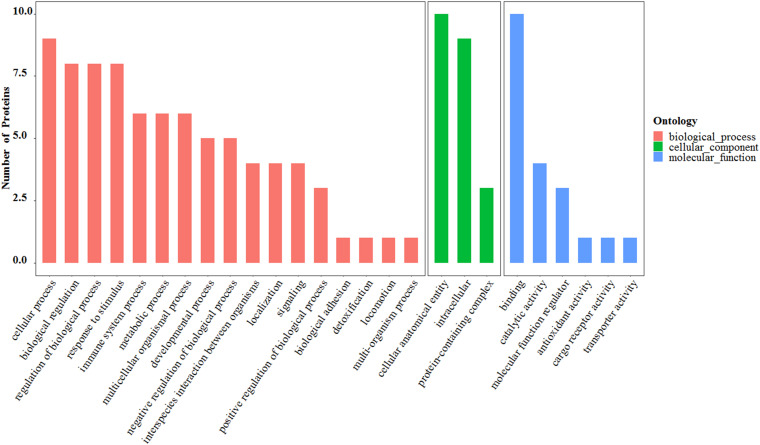
The Gene Ontology (GO) functional analysis of 10 differentially expressed salivary proteins.

The proteins found to be differentially expressed in the MB group showed significant enrichment of cellular and molecular processes involved in “*cellular process*,” “*biological regulation*,” “*regulation of biological process*,” “*response to stimulus*,” “*immune system process*,” “*cellular anatomical entity*,” “*intracellular*,” and “*binding*” (Figure 5). The Gene Ontology (GO) pathway mapping revealed that the most significantly regulated processes in the MB group were “*regulation of leukocyte mediated immunity*” and “*heat shock protein binding.*”

### Specific Indicator Genera Are Associated With GLOl by CCA Analysis

**FIGURE 6:**
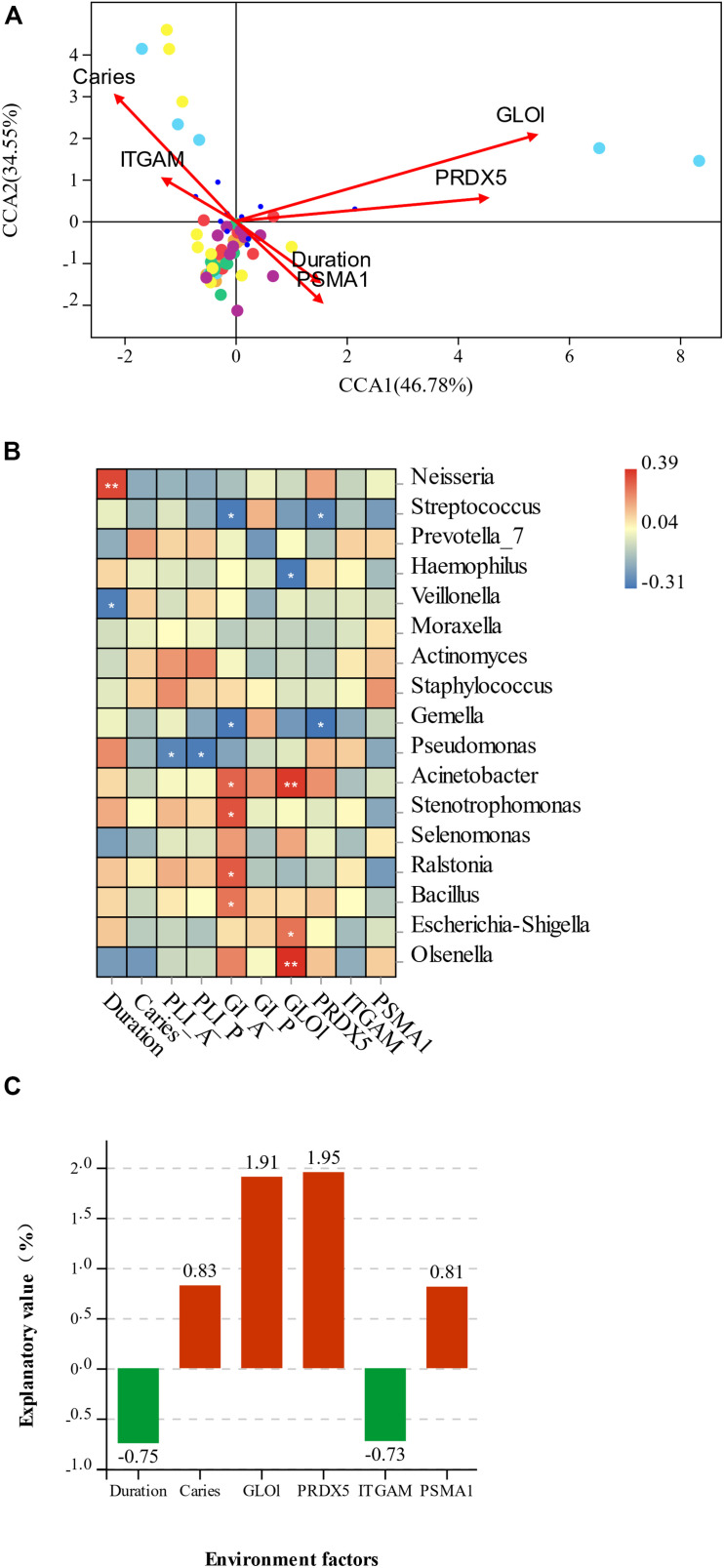
**(A)** Canonical correspondence analysis (CCA) ordination of the species and environmental factors in mouth-breathing (MB) and nose-breathing (NB) groups; **(B)** the Spearman correlation analysis of environmental factors and the most contributing species; **(C)** variance partitioning (%) analysis of environmental factors; *p < 0.05, **p < 0.001. The variable “Duration” represents the duration of mouth breathing; “caries” represents have caries or not, A/P represent anterior/posterior area.

CCA analysis of the association between the microbiota of two groups sample and differentially expressed salivary protein, duration of mouth breathing, and other characteristics (age and gender of participants, with or without caries, PlI and GI) showed that the abundance of *Acinetobacter*, *Escherichia–Shigella*, and *Olsenella* was positively related to GLOl, and the abundance of *Streptococus* and *Gemella* was negatively related to PRDX5. The duration of mouth breathing was positively associated with the abundance of *Neisseria* and negatively associated with *Veillonella*. The inflammation status of the anterior gingiva was positively related to *Acinetobacter*, *Stenotrophomonas*, *Ralstonia*, and *Bacillus* and negatively related to *Streptococcus* and *Gemella*. The relationships between environmental factors and the most contributing species and non-parametric Spearman correlation analysis are illustrated in Figures 6A,B. Variance partitioning analysis (VPA) showed that GLOl (explanatory value = 1.91%) and PRDX5 (explanatory value = 1.95%) had the greatest contribution to the oral–nasal–pharyngeal species distribution (Figure 6C), while caries (0.83%) and PSMA1 (0.81%) had a medium contribution. The duration of mouth breathing was the only significant factor with a high explanatory value (6.89%) contributing to microbiota at the genus level.

## Discussion

**FIGURE 7:**
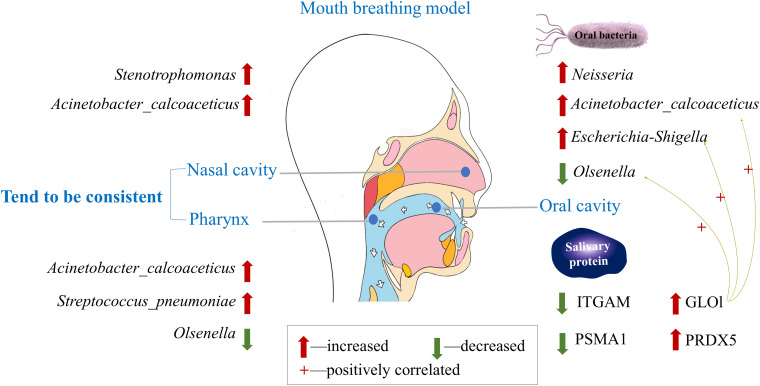
Understanding the alteration in oral–nasal–pharyngeal microbiota and salivary protein caused by mouth breathing.

The overall mode about species alteration of oral–nasal–pharyngeal sites and differentially expressed salivary proteins under mouth breathing are illustrated in Figure 7.

### Taxonomic Composition and Microbiota Diversity Changed Among Mouth-Breathing Children

There are few studies that exhaustively investigated the microbiota in subjects with mouth breathing. We present an overview of oral–nasal–pharyngeal microbiota profiles of 10 paired mouth-breathing and nose-breathing children. The dominant genera of samples from supragingival plaque, mucosa swabs of the anterior region, and unstimulated saliva in the oral cavity of the NB group (i.e., *Streptococcus*, *Actinomyces*, *Veillonella*, *Corynebacterium*, *Neisseria*, and *Leptotrichia*) are in general agreement with previous findings (Keijser et al., 2008; Gao et al., 2018). Likewise, the most abundant genera in nasal samples of the NB group (*Corynebacterium_1*, *Streptococcus*, *Dolosigranulum*, *Staphylococcus*, *Haemophilus*, and *Moraxella*) were commonly observed in a study and found that the microbiota was associated with both respiratory and gastrointestinal infections (Verhagen et al., 2020). Among the dominant genera, *Streptococcus* and *Neisseria* can produce acetaldehyde, which is affected by environmental conditions (aerobic or anaerobic) (Donati et al., 2016).

Alterations of the microbial composition and diversity in the oral–nasal–pharyngeal samples may be directly due to the change in environment; for example, mouth breathing could influence changes in humidification, temperature, pH, and oxygen level of the respiratory tract and oral cavity (Choi et al., 2016; Fischer et al., 2017). Indeed, several genera of aerobic bacteria (*Neisseria* and *Kingella*) had higher relative abundance, while anaerobic bacteria (*Leptotrichia* and *Selenomonas*) had lower relative abundance in the oral cavity. Ding and Schloss (2014) suggested that the community of oral cavity were the least stable, which may be shaped by an individual’s recent interactions with the environment, diet, medications, and overall health. Except for the environment, it is widely accepted that microbiota composition is significantly affected by the mechanism underlying microbial interference. Aerobic bacteria colonize different sites within the oral cavity and pharynx revealing close associations between species and tissues, which represent unique microbial ecological niches with specific physicochemical conditions (Donati et al., 2016). There is an inverse correlation in the oropharynx (*Firmicutes* and *Proteobacteria*) and in the nostril (*Firmicutes* and *Actinobacteria*), suggesting a potential antagonism between these groups (Lemon et al., 2010). The human nasal microbiota is highly variable and dynamic, often containing major pathogens such as *Staphylococcus aureus*, and other *Firmicutes* could produce bacteriocin to limit the growth of other nasal bacteria (Daniela et al., 2016). The higher diversity of oral–pharyngeal microbiota and alterations of bacteria community in mouth-breathing children may indulge some opportunistic pathogens.

In mouth-breathing children, opportunistic pathogens enriched with *Acinetobacter* in anterior supragingival plaque and *S. pneumoniae* in pharynx may uncover the potential mechanism of disease. *Acinetobacter* is a major cause of nosocomial infections (Almasaudi, 2018) and is also frequently detected in the oral microbiota of individuals with chronic periodontitis, poor oral hygiene, and cigarette use (Souto et al., 2014). *S. pneumoniae* is the most frequent colonizers of the pediatric nasopharynx and acts as a prerequisite for infectious diseases (Van der Poll and Opal, 2009). A previous study showed that pneumococcal carriage initiates an interleukin (IL)-17A-mediated immune response in nasopharyngeal adenoids, which might be associated with adenoidal hypertrophy that leads to mouth breathing (Huang et al., 2018). Increased *Stenotrophomonas* in nostrils is associated with chronic biofilm infections in patients with cystic fibrosis (Høiby et al., 2017) and was enriched in chronic rhinosinusitis patients without nasal polyps compared to healthy controls (Koeller et al., 2018). These aerobic pathogens increased under the environment with sufficient oxygen and may be the initiating factor of diseases induced by mouth breathing.

### Mouth Breathing Was Associated With Respiratory Health

The oral cavity and upper respiratory tract is colonized by specialized resident bacterial, viral, and fungal assemblages, which presumably play key roles in maintaining immune homeostasis (Feller et al., 2013), prevent potential pathogens from overgrowing and disseminating toward the lungs and may play a role as gatekeepers to respiratory health (Man et al., 2017). Accumulation of specific bacteria or distorted equilibrium between microbial immigration and microbial elimination in the respiratory tract is the precursor of respiratory disease. There are several studies to investigate the influence of microbiota on respiratory health. Iwai et al. (2012) found that HIV-infected patients with recurrent pneumonia exhibit relatively increased abundances of several known or suspected pathogenic organisms (oral cavity, *Bacteroides*, *Firmicutes*, and TM7 phyla; airway, *Proteobacteria*). Yang et al. (2019) found that the oral microbiome may play an important role in HIV-associated chronic obstructive pulmonary disease (COPD) pathogenesis, possibly by stimulating inflammation and promoting lung damage. Apart from COPD patients, immune maturation and allergy development (particularly asthma) also appear to be influenced by early changes in oral microbial composition (Dzidic et al., 2018). Microbial immigration from the oral cavity might be the significant source of the lung microbiome during health (Bassis et al., 2015). Among mouth-breathing children, the accumulation of opportunistic pathogens in the pharynx and oral cavity may be a serious threat for respiratory health through bacteria colonization, the interaction between flora, and other potential mechanism.

### The Potential Mechanism Underlying Oxidative Stress–Inflammation Caused by an Imbalance of the Oral–Pharyngeal Microecology

Alterations of salivary proteins in the MB group indicate a state of oxidative stress and inflammation caused by mouth breathing. The proteins with higher abundance, including GLOl (2.04-fold) and PRDX5 (1.62-fold), played an important role in the oxidative stress process. GLOl is an enzyme involved in the detoxification of methylglyoxal, which inhibits the growth of cells in organisms (Kalapos, 1999). Increased salivary GLOl suggests accumulation of methylglyoxal, while animal experiments showed that oral intake of methylglyoxal exacerbates Th2-mediated airway eosinophil infiltration by activation of the nuclear factor-kB/inducible nitric oxide synthase (NF-κB/iNOS)-dependent signaling pathway and positive regulation of NADPH oxidase 2 (NOX-2) and NADPH oxidase 4 (NOX-4) in the lung tissues of laboratory mice (Medeiros et al., 2020). An *in vitro* study had demonstrated that in *S. mutans*, GLOl functions in the detoxification of methylglyoxal, resulting in increased aciduricity (Korithoski et al., 2007), which may be the explanation of decreased oral pH among mouth-breathing children. PRDX5 is a thioredoxin peroxidase that reduces hydrogen peroxide, peroxynitrite, and alkyl hydroperoxides (Islinger et al., 2012). PRDX5 exerts a cytoprotective function against oxidative attacks induced by exogenous H_2_O_2_ (Walbrecq et al., 2020). Knoops et al. (2018) showed that extracellular human PRDX5 can activate a proinflammatory response. These results indicated oxidative damage with an increase in relative protein secretion into the saliva in mouth-breathing children. There are literatures suggesting that salivary oxidative damage products could generally reflect the content of plasma (Maciejczyk et al., 2019), which could enter saliva via gingival crevicular fluid (Siqueira Walter and Dawes, 2011), while mouth breathing could affect children’s general health (Flutter, 2006; Kuroishi et al., 2015). Some cytokines and bacterial products may lead to host immune reaction, and oral epithelial cells could produce diverse proinflammatory cytokines and chemokines (Groeger and Meyle, 2019).

While among the proteins with lower abundance, integrin alpha-M (also known as CD11b) is critical for leukocyte adhesion and migration for immune functions, small molecule-mediated activation of integrin CD11b/CD18 reduces inflammatory disease (Maiguel et al., 2011) and is associated with T-cell-mediated immune suppression by neutrophils after influenza infection (Tak et al., 2018). Another protein, PSMA1, which is involved in the ubiquitin–proteasome system (UPS), controls many cellular processes, and dysregulation of this fine-tuning is likely to induce cell death and promote inflammation (Brehm and Krüger, 2015). Accumulated evidence indicate that oxidative stress and inflammation are highly correlated and orchestrated to drive the pathophysiological procedure of various diseases-like liver diseases (Li et al., 2016), cardiovascular disease (Steven et al., 2019), type 2 diabetes mellitus and obesity (Picu et al., 2017), and pre-eclampsia (PE) (Tenório et al., 2019). There is also evidence indicating that respiratory disease is associated with abnormal inflammation and high oxidative stress, such as chronic obstructive pulmonary disease (COPD) (Ye et al., 2019) and cystic fibrosis (Livnat et al., 2010).

Growing evidence has suggested that the compositional and functional changes of the human microbiome are capable of regulating the development and function of the immune system via epigenetic mechanisms, which may break immune homeostasis and finally result in inflammation of immune associated tissue (Chen et al., 2017). Given the correlation between salivary proteins and specific bacteria in this study, the potential mechanisms of inflammation, including oxidative stress, upregulate expression of toll-like receptors (TLR2/4), immune-metabolic regulators (IRF3/5), and signature proinflammatory cytokines in human peripheral blood mononuclear cells (PBMCs), involving mitogen-activated protein kinase/nuclear factor-kB (MAPK/NF-κB)-dependent signaling, all of which have implications for metabolic inflammation (Akhter et al., 2019). Free bacterial toxins trigger increased circulating proinflammatory cytokines and a weakened immune system as well as cross-reactivity (molecular mimicry) between bacteria and self-antigens (Seymour et al., 2007). The host interacts with microbiota through proteins, metabolites, small molecules, and nucleic acids (Durmus et al., 2015; Guven-Maiorov et al., 2016), and further studies based on existing results are needed to investigate the effects and pathological mechanisms of mouth breathing.

There were some limitations to this study. The participants recruited in this study were not satisfyingly sufficient because of the strict inclusion criteria. We ensured the sample size meet the minimum standards and designed paired research with multisites samples to get more information precisely. Based on the comprehensive results, we grasped the general pattern of consequences that may be caused by mouth breathing. In this observational study, saliva secretion, children BMI, and vitamins and dietary supplements information were not included, and confounding factors may existed; it should be cautious to conclude the interactions between mouth breathing and the differences in microbiota and salivary proteins. Further studies about specific protein (e.g., GLOl) and redox state of saliva in longitudinal study and *in vitro* experiment to assess the proinflammatory effect of oxidative stress on oral epithelial cells and leukomonocyte were essential to verify the correlation. The mechanism underlying related inflammation and immunological processes also needs intensive study for better understanding mouth breathing and facilitating the search for biomarkers.

In conclusion, we first identified the alteration of composition and diversity of oral–nasal–pharyngeal microbiota among mouth-breathing children and discovered a matter of fact that species of nasal cavity and pharynx tended to be consistent. Some pathogens were enriched, including *Acinetobacter* in the anterior supragingival plaque, *Neisseria* in unstimulated saliva, *S. pneumoniae* in the pharynx, and *Stenotrophomonas* in the nostrils. Higher abundance of salivary GLOl and PRDX5, lower abundance of ITGAM and PSMA1, and the positive correlation with indicator species suggested oxidative stress and inflammatory state. Taken together, the results of this study provide meaningful information that contributes to our understanding of mouth breathing and relative respiratory disease. However, further studies, such as on biomarkers based on specific bacteria and salivary protein in a longitudinal study and the pathological mechanisms with inflammatory reactions, are still needed.

## Data Availability Statement

The datasets generated for this study can be found in the NCBI: GenBank accession number PRJNA642373. The RAW data and database search results of proteomics have been deposited to PRIDE from the ProteomeXchange consortium (PXD021106).

## Ethics Statement

The studies involving human participants were reviewed and approved by Ethics Committee of Guanghua School of (Ethics number: 2019-20; date of approval: 2019-3-1). Written informed consent to participate in this study was provided by the participants’ legal guardian/next of kin. Written informed consent was obtained from the individual(s), and minor(s)’ legal guardian/next of kin, for the publication of any potentially identifiable images or data included in this article.

## Author Contributions

HL, YH, and CF: conceptualization. CF, YH, and LG: methodology. HG and LG: validation and resources. CF: data analysis, writing—original draft preparation. YH, HL, LG, and HG: writing—review and editing. HL: supervision and project administration. HL and YH: funding acquisition. All authors have read and agreed to the published version of the manuscript.

## Conflict of Interest

The authors declare that the research was conducted in the absence of any commercial or financial relationships that could be construed as a potential conflict of interest.

## Footnotes

1 https://www.hmpdacc.org/doc/HMP_MOP_Version12_0_072910.pdf

2 http://proteomecentral.proteomexchange.org
